# Strain of Synechocystis PCC 6803 with Aberrant Assembly of Photosystem II Contains Tandem Duplication of a Large Chromosomal Region

**DOI:** 10.3389/fpls.2016.00648

**Published:** 2016-05-12

**Authors:** Martin Tichý, Martina Bečková, Jana Kopečná, Judith Noda, Roman Sobotka, Josef Komenda

**Affiliations:** ^1^Laboratory of Photosynthesis, Institute of Microbiology, Academy of Sciences of the Czech Republic, Center AlgatechTřeboň, Czech Republic; ^2^Faculty of Science, University of South BohemiaČeské Budějovice, Czech Republic

**Keywords:** *Synechocystis* 6803, chlorophyll, photosystem I, photosystem II assembly, large tandem duplication

## Abstract

Cyanobacterium *Synechocystis* PCC 6803 represents a favored model organism for photosynthetic studies. Its easy transformability allowed construction of a vast number of *Synechocystis* mutants including many photosynthetically incompetent ones. However, it became clear that there is already a spectrum of *Synechocystis* “wild-type” substrains with apparently different phenotypes. Here, we analyzed organization of photosynthetic membrane complexes in a standard motile Pasteur collection strain termed PCC and two non-motile glucose-tolerant substrains (named here GT-P and GT-W) previously used as genetic backgrounds for construction of many photosynthetic site directed mutants. Although, both the GT-P and GT-W strains were derived from the same strain constructed and described by Williams in 1988, only GT-P was similar in pigmentation and in the compositions of Photosystem II (PSII) and Photosystem I (PSI) complexes to PCC. In contrast, GT-W contained much more carotenoids but significantly less chlorophyll (Chl), which was reflected by lower level of dimeric PSII and especially trimeric PSI. We found that GT-W was deficient in Chl biosynthesis and contained unusually high level of unassembled D1-D2 reaction center, CP47 and especially CP43. Another specific feature of GT-W was a several fold increase in the level of the Ycf39-Hlip complex previously postulated to participate in the recycling of Chl molecules. Genome re-sequencing revealed that the phenotype of GT-W is related to the tandem duplication of a large region of the chromosome that contains 100 genes including ones encoding D1, Psb28, and other PSII-related proteins as well as Mg-protoporphyrin methylester cyclase (Cycl). Interestingly, the duplication was completely eliminated after keeping GT-W cells on agar plates under photoautotrophic conditions for several months. The GT-W strain without a duplication showed no obvious defects in PSII assembly and resembled the GT-P substrain. Although, we do not exactly know how the duplication affected the GT-W phenotype, we hypothesize that changed stoichiometry of protein components of PSII and Chl biosynthetic machinery encoded by the duplicated region impaired proper assembly and functioning of these multi-subunit complexes. The study also emphasizes the crucial importance of a proper control strain for evaluating *Synechocystis* mutants.

## Introduction

Cyanobacteria represent excellent model organisms for photosynthesis research as they perform oxygenic photosynthesis similar to that in algae and plants while having much simpler cellular organization. Unlike the other photosynthetic bacteria they possess both types of reaction center and oxidize water to molecular oxygen by Photosystem II (PSII) and reduce NADP by Photosystem I (PSI). For both pigment-protein complexes the high resolution crystal structures are available, mostly thanks to thermophilic cyanobacteria with very stable complexes ideal for crystallographic analysis (Jordan et al., 2001; Ferreira et al., 2004; Umena et al., 2011).

PSII is the multi-subunit membrane complex that utilizes light energy to catalyze oxidation of water. For this process to occur the inner symmetrically located antennae CP47 and CP43 containing chlorophyll (Chl) and β-carotene deliver absorbed light energy to a pair of reaction center (RCII) subunits called D1 and D2, which bind the cofactors involved in primary charge separation. There are also around 13 small, mostly single helix trans-membrane subunits mostly bound at the periphery of D1, D2, CP47, and CP43 (Ferreira et al., 2004; Guskov et al., 2009; Umena et al., 2011). The lumenal part of the complex is the binding site for the PsbO, PsbU, and PsbV extrinsic subunits (reviewed in Roose et al., 2007) which optimize the environment for the CaMn_4_O_5_ cluster extracting electrons from water (Ferreira et al., 2004; Guskov et al., 2009; Umena et al., 2011).

Information on the biogenesis of cyanobacterial PSII is less complete and mostly comes from characterization of this process in the cyanobacterium *Synechocystis* PCC 6803 (hereafter *Synechocystis*). This cyanobacterial strain is able to grow in the presence of glucose even without functional PSII and is therefore appropriate for constructing site-directed mutants affected in its assembly (Williams, 1988). Existing data indicate that each large Chl-protein initially forms a pre-complex (module) containing neighboring low-molecular-mass polypeptides plus pigments and cofactors. These modules are afterwards combined in a step-wise fashion initially into the PSII reaction center (RCII) complex consisting of D1 and D2 modules, then CP47 module is attached forming a complex called RC47 (Boehm et al., 2011, 2012). Finally the PSII core complex is completed after binding of the CP43 module (Boehm et al., 2011). The light-driven assembly of the oxygen-evolving CaMn_4_O_5_ cluster occurs afterwards and is assisted by the lumenal extrinsic proteins (Nixon et al., 2010). Accessory factors, such as Ycf39, Ycf48, Psb27, Psb28, and others, associate transiently with PSII at specific stages of assembly but their functions remain unclear (Komenda et al., 2012b).

Availability of high-throughput sequencing techniques allowed genomic sequence comparison among various WT-variants (substrains) of *Synechocystis* used in many laboratories and revealed inherent sequence variability among them. The history of the *Synechocystis* WT-variants has been described recently (Morris et al., 2014). The original motile Pasteur Culture Collection 6803 strain has been used to generate a non-motile, glucose tolerant strain (Williams, 1988). This strain has been disseminated to several laboratories around the world, resulting in many “wild-type” substrains.

In the present study we compared organization of photosynthetic membrane complexes with emphasis on PSII assembly in three *Synechocystis* substrains, two of them variants of the Williams glucose-tolerant strain. The comparison showed that the level and assembly state of PSII (and PSI) in the glucose-tolerant strain obtained from the laboratory at Imperial College London was similar to that in the standard motile Pasteur Culture collection strain (PCC). In contrast, the glucose tolerant strain originating from the strain used in the Arizona lab of Wim Vermaas showed aberrant assembly of PSII, much lower level of PSI related to the decreased synthesis of Chl and high content of carotenoids. Sequencing of the latter strain showed that its genome contains a tandem duplication of a large chromosomal region, most probably responsible for the observed phenotype.

## Experimental procedures

### Strains, their origin, construction, and cultivation

The strains used in this study were the motile, glucose sensitive strain of *Synechocystis* sp. PCC 6803 from the Pasteur collection, and two glucose-tolerant strains (Williams, 1988): GT-P obtained from the laboratory of Prof. Peter Nixon at Imperial College London and GT-W obtained from the laboratory of Prof. Wim Vermaas at Arizona State University. Frozen stocks of GT-P from 2002 and GT-W from 1999 were used for characterization and sequencing. The Psb28-lacking strains were transformed using genomic DNAs isolated from a previously constructed Psb28-less strain (Dobáková et al., 2009). *Synechocystis* strains were grown autotrophically in a rotary shaker under irradiance of 40 μmol photons m^−2^ s^−1^ at 30°C in liquid BG11 medium (Rippka et al., 1979). All experiments and measurements with cells were performed at least in triplicate and typical results are shown in figures.

### Cell absorption spectra and determination of Chl content

Absorption spectra of whole cells were measured at room temperature using a Shimadzu UV-3000 spectrophotometer (Kyoto, Japan). To determine Chl levels, pigments were extracted from cell pellets with 100% methanol and the Chl concentration was determined spectroscopically (Porra et al., 1989).

### Determination of chromosomes number by flow cytometry

Single colonies of GT-P and GT-W strains were inoculated in 50 ml of BG11 medium and grown under standard conditions until they reached a final OD_730nm_ value 0.3–0.5. Two milliliters of cells were collected by centrifugation at 8000 × g for 5 min and washed once with fresh media. Collected cells were fixed with 1 ml of 70% ethanol and incubated for 1 h at room temperature. In order to remove residual fixation solution, cells were washed twice with PBS buffer and resuspended in 100 μl of the same buffer. One microliter of RNAase A was added and samples were incubated for 1 h at 37°C. After enzymatic degradation of RNA, 900 μl of PBS were added, and cells were stained for 20 min in darkness with the cell permeant fluorochrome SYBR Safe (Life Technologies), used in a 1:10,000 dilution of commercial stock. To prepare the reference, with one chromosome per cell a single colony of *Escherichia coli (E. coli)* strain BL-21 was inoculated in 2 ml of M9 minimal media. The culture was grown overnight at 37°C and then supplemented with 20 μg ml^−1^ of chloramphenicol. After 1.5 h of growth in the presence of antibiotic, cells were harvested by centrifugation at 8000 × g for 5 min and fixed and stained by the same method used for *Synechocystis* cells. *Synechocystis* and *E. coli* stained cells were loaded on an APOGEE cytometer (Apogee Flow Systems), and at least 20,000 events were recorded. Samples were excited using a 488 nm laser and the fluorescence emission was collected by a detector with a 530/30 nm filter. The chromosome copy number of *Synechocystis* strains was estimated using the chloramphenicol-treated *E. coli* culture as a standard.

### Analysis of pigments by HPLC

For quantitative determination of Chl precursors, three milliliters of culture at OD_750nm_ = 0.5–0.6 was spun down and resuspended in 20 μL of water. Pigments were extracted with an excess of 70% methanol/30% water, filtrated and immediately analyzed via HPLC (Agilent-1200). Separation was carried out on a reverse phase column (ReproSil pur 100, C8, 3 μm particle size, 4 × 150 mm, Watrex) with 35% methanol and 15% acetonitrile in 0.25 M pyridine (solvent A) and 50% methanol in acetonitrile as solvents B. Pigments were eluted with a gradient of solvent B (40%–52% in 5 min) followed by 52–55% of solvent B in 30 min at a flow rate of 0.8 ml min^−1^ at 40°C. Eluted pigments were detected by two fluorescence detectors set at several different wavelengths to detect all Chl precursors from coproporphyrin(ogen) III (Copro III) to monovinyl-chlorophyllide (Chlide); for details see Kopečná et al. (2015)

### 2D electrophoresis, immunodetection, and protein radiolabeling

Membrane and soluble protein fractions were isolated from 50 ml of cells at OD_750nm_ ~0.4 according to Dobáková et al. (2009) using buffer A (25 mM MES/NaOH, pH 6.5, 5 mM CaCl_2_, 10 mM MgCl_2_, 25% glycerol). Isolated membrane complexes (0.25 mg ml^−1^ Chl) were solubilized in buffer A containing 1% n-dodecyl-β-D-maltoside and analyzed either by blue-native (BN) or by clear-native (CN) PAGE at 4°C in a 4–14% gradient polyacrylamide as described in Komenda et al. (2012a). The protein composition of the complexes were analyzed by electrophoresis in a denaturing 12–20% linear gradient polyacrylamide gel containing 7 M urea (Komenda et al., 2012a). Proteins separated in the gel were stained by SYPRO Orange and the gel was either dried and exposed on a Phosphorimager plate or blotted onto a PVDF membrane. Membranes were incubated with specific primary antibodies and then with secondary antibody-horseradish peroxidase conjugate (Sigma, St. Louis, USA). The primary antibodies used in this study were raised in rabbits against: (i) residues 58–86 of the spinach D1 polypeptide; (ii) residues 311-322 of Ycf39 (Knoppová et al., 2014); and (iii) the last 15 residues of *Synechocystis* Psb28 (Dobáková et al., 2009). Antibody against *Synechocystis* ferrochelatase was kindly provided by Prof. Annegret Wilde (Albert Ludwigs Universität, Freiburg) and antibody against barley Mg-protoporphyrin methyl ester oxidative cyclase was kindly provided by Prof. Poul Erik Jensen (University of Copenhagen).

For protein and Chl labeling, the cells were incubated with [^14^C]glutamate for 30 min as described in Kopečná et al. (2012). After separation of labeled proteins by CN PAGE in the first dimension and by SDS PAGE in the second dimension the 2D 18% polyacrylamide gel was stained by SYPRO Orange, scanned for fluorescence and dried. The gel was exposed on a Phosphorimager plate overnight, scanned by Storm and, for evaluation of Chl labeling, the image was quantified by ImageQuant 5.2 software (all from GE Healthcare, Vienna, Austria).

### Genome re-sequencing and mapping

*Synechocystis* re-sequencing was performed commercially at the Gene Profiling Facility, Princess Margaret Hospital, Toronto. One hundred base paired-end sequencing was performed on a Illumina HiSeq 2000 system (12 samples per lane). Raw paired reads were mapped to the GT-Kazusa sequence using Geneious 7.0 software (http://www.geneious.com; Kearse et al., 2012). Only variants with a higher than 60% frequency were considered. Alternatively, read sequences were assembled *de novo* with the Geneious 7.0 software before mapping to the reference.

## Results

### Three variants of the *Synechocystis* wild type strain: their history and organization of photosynthetic complexes

We compared three *Synechocystis* strains that are used in our laboratory for construction of various, mostly photosynthetic mutants. While the glucose sensitive motile strain, designated here PCC, was directly obtained from Pasteur Culture Collection, the other two strains originated from the glucose-tolerant Williams strain (Williams, 1988). The first one, which we named GT-P, was first grown in the Dupont laboratory of Bruce Diner from which it was transferred to Imperial College (London) and later to our laboratory in Třeboň (Institute of Microbiology, Czech Academy of Sciences). The second one, named GT-W, came from the laboratory of Wim Vermaas (Arizona State University).

**Figure 1:**
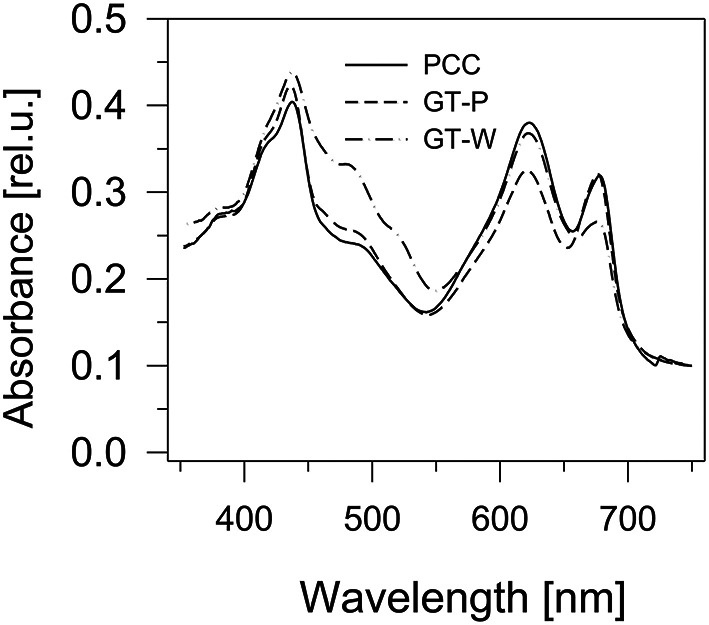
**Whole cell absorption spectra of three ***Synechocystis*** strains PCC, GT-P, and GT-W**. The spectra were measured by Shimadzu UV3000 spectrophotometer and were normalized for absorbance of 0.1 at 750 nm.

Comparison of absorption spectra of the three strains grown autotrophically showed a large similarity between the PCC and GT-P, with the only apparent difference being a higher phycobilisome content (maximum at 625 nm) in PCC (Figure 1). On the other hand, the GT-W strain contained much more carotenoids and less Chl than both previously mentioned strains. Interestingly, when both strains were grown in the presence of 5 mM glucose, in both strains the amount of carotenoids decreased but the level of Chl decreased only in GT-P while in GT-W it increased (Figure S1).

**Figure 2:**
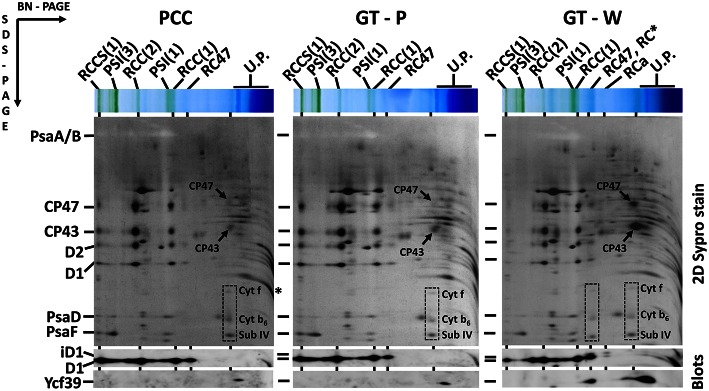
**Organization of photosynthetic membrane complexes in PCC, GT-P, and GT-W strains assessed by 2D blue-native/SDS-PAGE in combination with immunoblotting**. Membrane proteins of thylakoids isolated from each strain were separated by 4–14% blue-native PAGE, and then in the second dimension by 12–20% SDS-PAGE. The 2D gel was stained by SYPRO Orange and then blotted to a PVDF membrane. The D1 and Ycf39 proteins were detected by specific antibodies (Blots). Unassembled CP47 and CP43 are designated by arrows and subunits of cyt b_6_–f complex (Cyt f, cyt b_6_, and subunit IV in order from top to bottom) are boxed. Other designations: RCCS1, PSI–PSII supercomplex; RCC(2), and RCC(1), dimeric, and monomeric PSII core complexes; PSI(3) and PSI(1), trimeric and monomeric PSI; RC47, PSII core complex lacking CP43; RC^*^ and RCa, PSII reaction center complexes lacking CP47 and CP43; U.P., unassembled proteins. Arrows indicate unassembled forms of CP47 and CP43 very abundant in GT-W. Each loaded sample contained 5 μg of Chl.

The differences in cellular absorption spectra of autotrophic cultures were also reflected by differences in the content of photosynthetic membrane complexes assessed by 2D blue-native/SDS-PAGE (Figure 2). In this respect PCC and GT-P were very similar containing the majority of PSI and PSII as trimers and dimers, respectively, and relatively low levels of the monomeric PSII core complex and CP43-less PSII (RC47). The strains also contained only very low amounts of unassembled CP47 and CP43 and no detectable antenna-less RCII complexes. In contrast, unusually high amounts of unassembled CP47 and especially CP43 were detected in the GT-W strain. Immunoblotting also revealed accumulation of RCII complexes and a concomitant several fold increase in the amount of Ycf39 detected as a component of a larger of two RCII complexes (RCII^*^) and especially as the unassembled complex with Hlips (Knoppová et al., 2014). Moreover, the levels of PSI trimer and PSII dimers were similar to the levels of their monomeric forms. Finally, cytochrome b_6_f complex was present in PCC and GT-P almost exclusively as a monomer while almost equal amounts of dimer and monomer was detected in GT-W. In summary, the strain GT-W showed unusual pigment-protein composition different from the other two strains suggesting aberrant assembly of photosynthetic complexes, namely PSII, and their lower cellular content.

### Chlorophyll biosynthesis is strongly affected in GT-W

**Figure 3:**
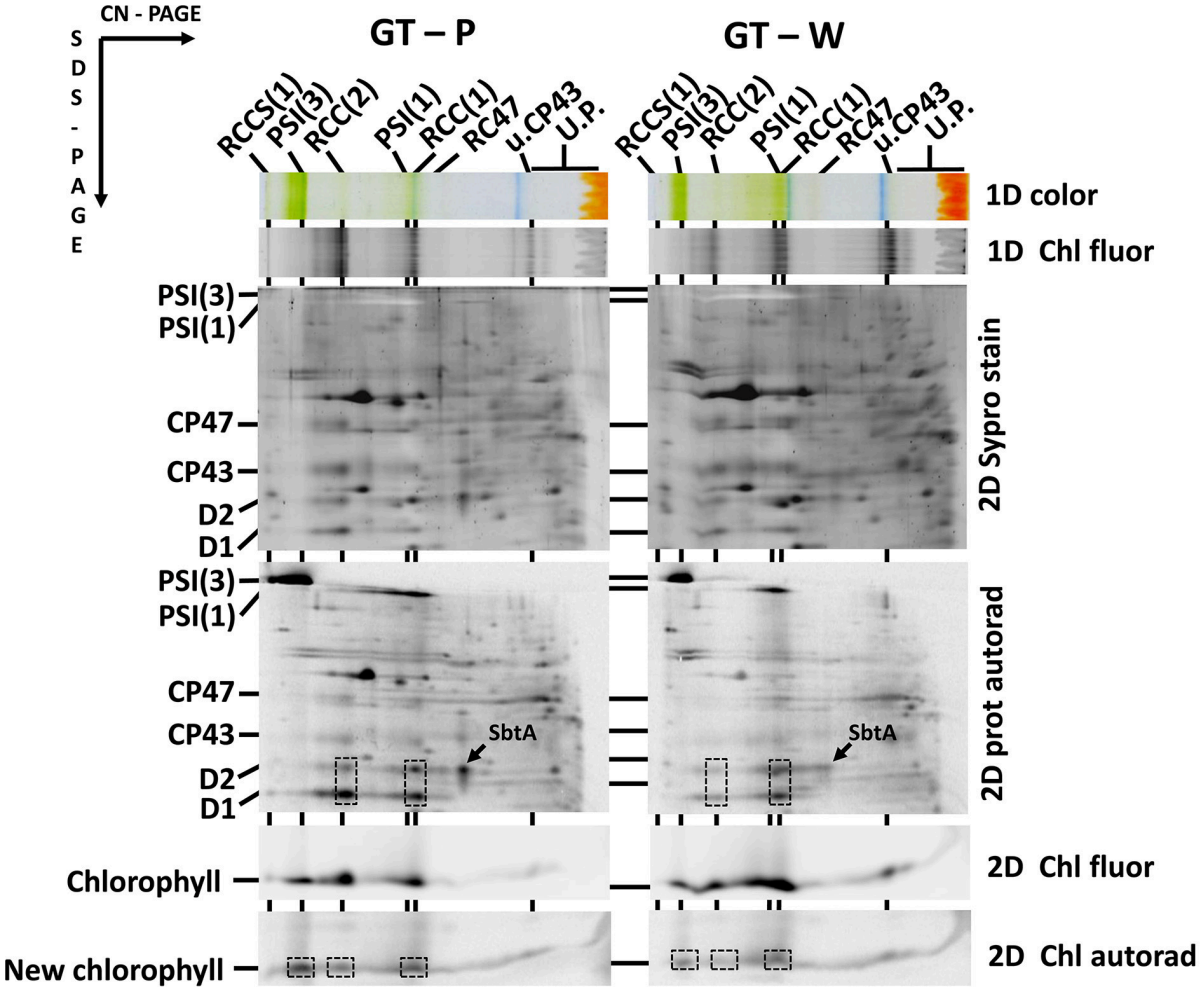
**Radiolabeling of membrane proteins and chlorophyll in the GT-P and GT-W strains**. Cells of each strain were radiolabeled by [^14^C]glutamate. Membrane proteins and pigments were then separated by 2D clear-native/SDS-PAGE. Pigment-protein complexes were separated by 2D CN/SDS-PAGE. The first dimension 4-14% native gel was photographed (1D color) and scanned for fluorescence by LAS 4000 (1D Chl fluor). The second dimension 18% SDS PAGE gel was stained with SYPRO (2D SYPRO stain) and scanned for Chl fluorescence (2D Chl fluor). Radioactivity in proteins (2D prot autorad) and in released Chl (2D Chl Autorad) in dried gels was detected by Phosphoimager. The overall signal of the labeled Chl in GT-W quantified by Image Quant software was 37 ± 9% of that in GT-P. The value represents a mean ± SD from three independent measurements. The most important differences between the strains in the amounts of labeled protein (monomeric and dimeric PSII complexes) and Chl (PSI trimer and monomer and PSII dimer) are emphasized by boxes. The labeling of SbtA is also designated by an arrow. Each loaded sample contained 5 μg of Chl. All designations used are described in Figure 2.

As the trimeric PSI complex is the main sink for newly produced Chl molecules in *Synechocystis* (Kopečná et al., 2012), the low PSI level in cells of GT-W should be reflected by changes in Chl metabolism. To assess the rate of *de novo* Chl formation we labeled cells of both GT strains using [^14^C]glutamate and analyzed Ch-binding membrane complexes using 2D clear-native/SDS-PAGE. In order to separate free pigments from proteins, we performed 2D protein analysis in the 18% gel, which was briefly stained by SYPRO Orange, scanned for Chl and SYPRO fluorescence and then immediately dried up to retain maximal amounts of free pigments in the gel. In agreement with work of Kopečná et al. (2012) most of the labeled Chl in GT-P was bound to trimeric PSI. However, in GT-W the signals of labeled Chl incorporated into the PSI trimer and PSII dimer were much weaker (Figure 3, lower boxes). Labeling in the abundant PSI monomer was also lower in GT-W. Overall, the total signal of labeled Chl in GT-W was <40% of that in GT-P, which demonstrated a significantly reduced rate of *de novo* Chl formation in the GT-W substrain. Since [^14^C]glutamate also labeled proteins, autoradiogram of the whole gel provided information about synthesis of membrane proteins. GT-W showed a typical protein labeling pattern with intensive labeling of D1 and much lower labeling of D2, CP43, and CP47 in both PSII core monomers and dimers. In contrast, the intensity of D1 and D2 labeling in the PSII core complexes of GT-W was similar but labeling of both proteins in the PSII dimer was much lower than in the monomer and in GT-P (Figure 3, middle boxes). Interestingly, the labeling of SbtA, a bicarbonate transporter, was also much lower in GT-W indicating an inability of the strain to actively induce import of anorganic carbon for CO_2_ fixation (Figure 3, arrows).

**Figure 4:**
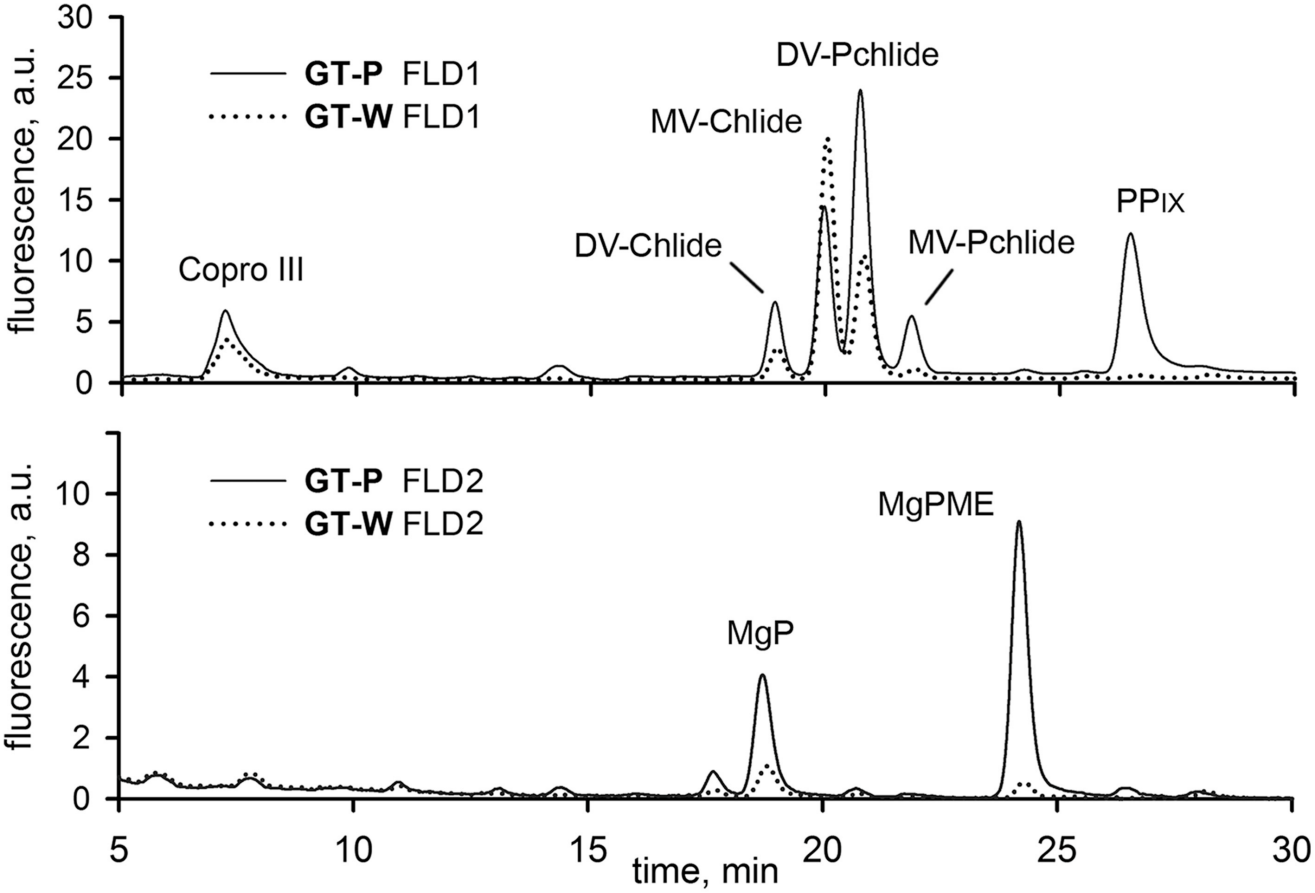
**HPLC chromatograms of chlorophyll biosynthesis precursors in GT-P and GT-W strains**. Cells of both strains were extracted with 70% methanol and Chl precursors were quantified by HPLC equipped with two fluorescence detectors (FLD1 and FLD2). Each detector was set to wavelengths selected to detect indicated Chl precursors (see Experimental Procedures). Copro III, coproporhyrin(ogen) III; DV-Chlide, divinyl chlorophyllide; DV-Pchlide, divinyl protochlorophyllide; PP_*IX*_, protoporphyrin IX; MgP, Mg-protoporphyrin IX; MgPME, Mg-protoporphyrin methylester; MV-Chlide, monovinyl chlorophyllide; MV-Pchlide, monovinyl protochlorophyllide.

To further support the conclusion about inhibition of Chl biosynthesis in GT-W, we compared levels of later Chl biosynthesis precursors in GT-P and GT-W (Figure 4). HPLC analysis confirmed that in GT-W the levels of most intermediates were strongly decreased. Only the level of coproporhyrin(ogen) III (copro III) in GT-W was rather similar to that in GT-P suggesting either a block in the subsequent formation of protoporphyrinogen IX and protoporphyrin IX (PP_*IX*_) or, alternatively, a fast consumption of PP_*IX*_ by the heme branch of the tetrapyrrole biosynthesis pathway.

### Genomic sequencing revealed duplication of a large part of GT-W chromosome

**Figure 5:**
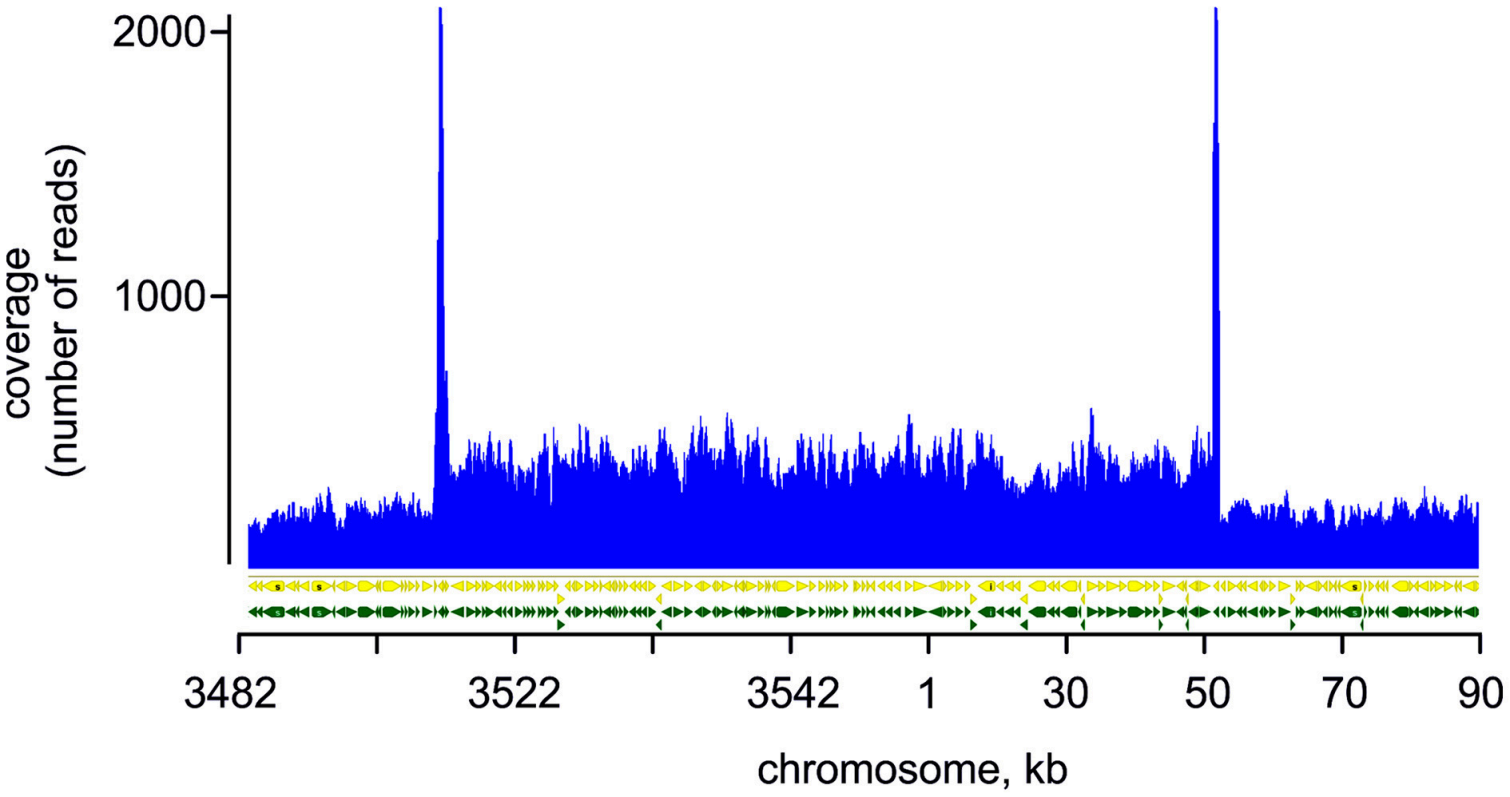
**Mapping of reads on the duplicated region of GT-W chromosome**. Paired-end reads from Illumina sequencing of the GT-W genome mapped on the reference *Synechocystis* genome (see Experimental Procedures). The read coverage is roughly doubled in the chromosomal region 3520 kb–50 kb; peaks at the border of this region are an artifact of the mapping of reads originating probably from transponase genes located on plasmids.

To explore the genetic basis for the observed aberrant assembly of photosynthetic complexes and decreased Chl biosynthesis, we performed whole genome re-sequencing of both GT strains on a commercial basis, using the Illumina HiSeq platform. Although, the sequence of the original Williams strain is not available, it can be interpolated from the published sequences of the GT strains. Our sequencing supports a common origin of both GT-P and GT-W strains from the Williams GT strain. The GT-P and GT-W strains shared one mutation while containing one and four additional specific mutations respectively (Table 1). In addition to the point mutations detected directly by mapping of the reads to the reference sequence, we observed that one part of the GT-W chromosome exhibited approximately twofold coverage of reads. Interestingly, this large 110 kbp region was bordered by ISY100 transposases, sll0431 and sll1397, sharing 100% identity at the DNA level (Figure 5, Table S1). It indicates that this part of the genome was duplicated, possibly in the form of a tandem repeat. Indeed, the presence of such tandem duplication was confirmed by PCR with outbound primers from the beginning and end of the duplication (Figure S2). The duplicated region contained 100 genes (Table S1) including those encoding PSII components (D1 protein) and its assembly factors (e.g., Psb28), which could possibly be related to the aberrant PSII assembly observed in the GT-W strain.

**Table 1:** **List of specific mutations in GT-P and GT-W strains**.

**Base position**	**Type**	**Mutated nucleotide**	**Amino acid change**	**Gene ID**	**Annotation**	**Gene product**
**GT-P**						
488230	SNP	T→G	F255C	*slr1609*	*fadD*	long-fatty-acid CoA ligase
842060	SNP	C→T	R186Q	*sll1799*	*rpl3*	50S ribosomal protein L3
**GT-W**						
488268	SNP	G→A	V268I	*slr1609*	*fadD*	long-fatty-acid CoA ligase
842060	SNP	G→T	R186Q	*sll1799*	*rpl3*	50S ribosomal protein L3
2354038	Del	5326 bp del	E283Q	*slr0364*	*swmB*	cell surface protein
2780356	SNP	G→C	E283Q	*slr0906*	*psbB*	CP47 protein of PSII
3297450	SNP	C→T	L216 silent	*sll1367*	–	hypothetical protein

*Base position refers to the GT-Kazusa sequence*.

**Figure 6:**
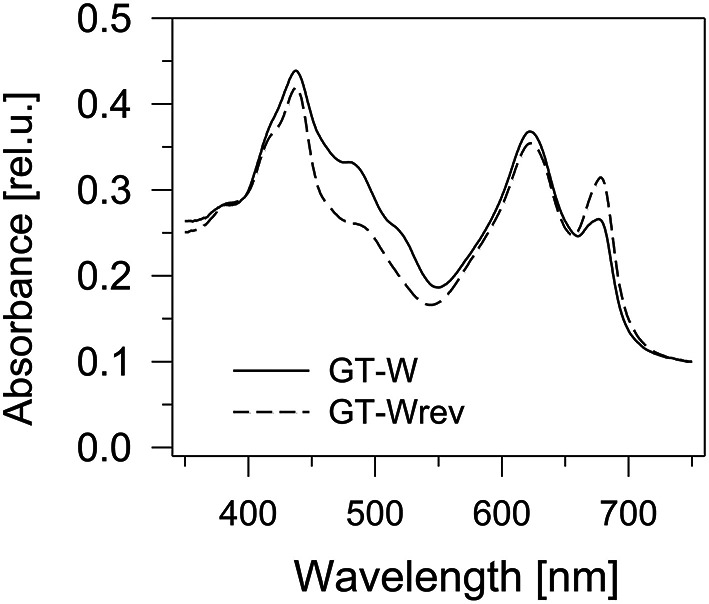
**Whole cell absorption spectra of the GT-W strain and its revertant GT-Wrev**. The spectra were measured by Shimadzu UV3000 spectrophotometer and normalized for absorbance of 0.1 at 750 nm.

**Figure 7:**
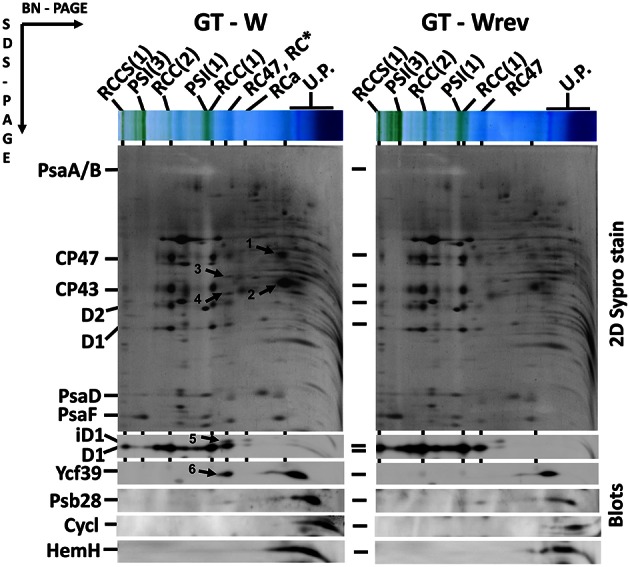
**Organization of photosynthetic membrane complexes in the GT-W strain and its revertant GT-Wrev assessed by 2D blue-native/SDS-PAGE in combination with immunoblotting**. Membrane proteins of thylakoids isolated from each strain were separated by 4–14% blue-native PAGE, and then in the second dimension by 12–20% SDS-PAGE. The 2D gel was stained by SYPRO Orange and then blotted to a PVDF membrane. The D1, Ycf39, Psb28, Cycl, and HemH proteins were detected by specific antibodies (Blots). Designations as in Figure 2. Arrows indicate unassembled forms of CP47(1) and CP43(2), the Ycf48 (3), and Ycf39 (4, 6) assembly factors, and an intermediate form of D1 (5) in RCII^*^ complex, which are all much more abundant than in GT-Wrev. Each loaded sample contained 5 μg of Chl.

Interestingly, the characteristics of the GT-W strain spontaneously changed when repeatedly restreaked on plates without glucose and under moderate light conditions (30 μmol photons s^−1^ m^−2^) for several months. The strain gradually attained a pigment composition similar to GT-P (Figure 6) and also the pattern of membrane protein complexes became similar to that in GT-P with disappearance of most of the unassembled CP43, CP47, and RCII^*^ and an increase in the level of PSI trimer (Figure 7). This strain also contained lower levels of Ycf39, Psb28, HemH, and Cycl in comparison with GT-W (Figure 7). PCR analysis showed that this revertant strain, designated GT-Wrev, lost the duplication (Figure S2) indicating that it is responsible for most of the Chl-deficient phenotype of GT-W.

**Figure 8:**
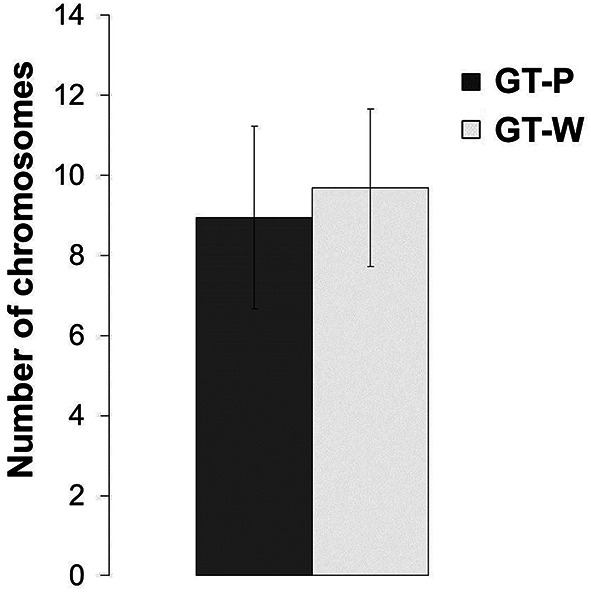
**Number of chromosomes detected in the strains GT-P and GT-W**. Calculation was done by comparison of the SYBR Safe fluorescence detected by flow cytometry in the both *Synechocystis* strains, using chloramphenicol-treated *E. coli* BL-21 culture as a reference.

Since the observed duplication of part of the genome could be a compensatory mechanism for a lower number of genome copies in the GT-W strain, we also compared the number of chromosomes in both strains. DNA content was assessed using flow cytometry after staining by SYBR Safe and the copy-number calibrated using *E. coli* cells (see Experimental Procedures). Figure 8 shows that both strains similarly contain 7–11 chromosome copies per cell.

### Psb28 deletion mutant constructed in the GT-W background shows more pronounced phenotype

**Figure 9:**
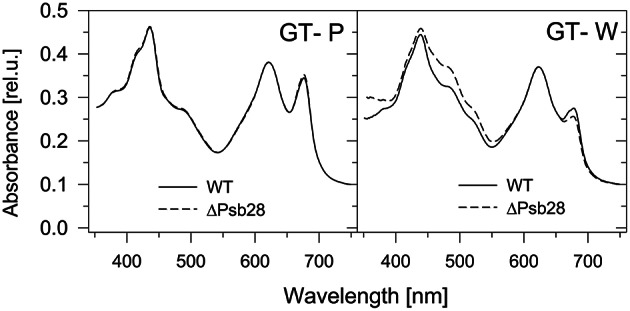
**Whole cell absorption spectra of the Psb28-deletion mutants in comparison with their background strains GT-P and GT-W used for their construction**. The spectra were measured by Shimadzu UV3000 spectrophotometer and normalized for absorbance of 0.1 at 750 nm.

**Figure 10:**
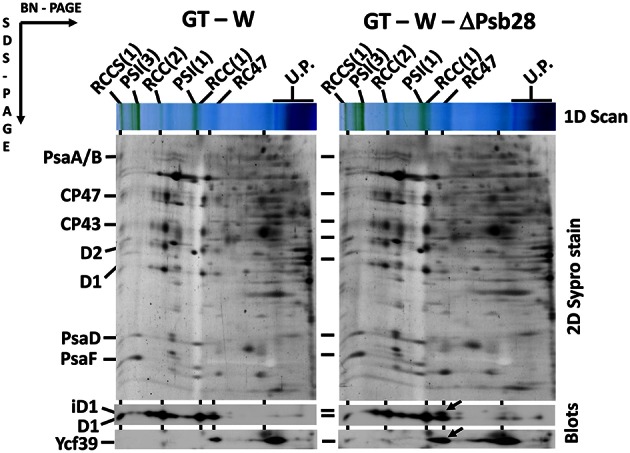
**Organization of photosynthetic membrane complexes in the GT-W strain and Psb28-less mutant constructed in this background**. Thylakoid proteins isolated from each strain were separated by 4–14% blue-native-PAGE, and then in the second dimension by 12–20% SDS-PAGE. The 2D gel was stained by SYPRO Orange and then blotted to a PVDF membrane. The D1 and Ycf39 proteins were detected by specific antibodies (Blots). Designations as in Figure 2. Arrows indicate intermediate forms of the D1 protein and the Yc39 assembly factor present in the RCII^*^ complex, which is more abundant in the Psb28-less strain. Each loaded sample contained 5 μg of Chl.

Previously, Dobáková et al. (2009) has shown a partial Chl deficiency in the Psb28-less strain, causing inhibition of CP47 and PSI synthesis. This strain was constructed in the GT-W background. In contrast, a subsequent study of Sakata et al. (2013) has not confirmed this phenotypic manifestation and the Psb28-less mutant, which has been constructed in WT background closely related to our GT-P strain and has not exhibited lower cellular Chl level. Since the different phenotypes of the mutants could be caused by a different genetic background of the mutants, we tested this possibility by deleting the *psb28* gene in both the GT-P and GT-W strains. *In vivo* absorption spectra of both mutants were then compared with the original control strains (Figure 9). The spectrum of the mutant constructed in the GT-P background was indistinguishable from the control, while the GT-W-based mutant showed a lower Chl level as well as much more carotenoids than the control. We also compared the composition of the membrane protein complexes of GT-W and the derived Psb28-less mutant (Figure 10). The mutant contained higher levels of the RCII complexes and Ycf39, the PSII assembly factor protein which has been proposed to participate in the Chl recycling (Knoppová et al., 2014). The data supported the idea that the differences observed in the two above mentioned studies (Dobáková et al., 2009; Sakata et al., 2013) were caused by the different WT background in which the mutants were constructed.

## Discussion

During our previous studies dealing with the biogenesis of photosynthetic membrane complexes in *Synechocystis* we characterized dozens of mutants constructed in different background strains originating from various laboratories. We noticed that mutants constructed in background strains coming from the London laboratory of Peter Nixon and the Arizona laboratory of Wim Vermaas had quite different phenotypes although they both originated from the glucose-tolerant Williams substrain (Williams, 1988; Morris et al., 2014). Under photoautotrophic conditions GT-W grew much slower than GT-P (not shown) and the cultures appeared quite different with regard to their cellular spectra, pigment content, and composition of membrane protein complexes. GT-P was in this respect very similar to the glucose sensitive motile PCC strain with minimal amount of unassembled or partially assembled PSII components. GT-W was much more yellowish and this also corresponded to higher carotenoid and lower Chl levels (Figure 1). Moreover, this was also accompanied by aberrant composition of Chl-binding complexes. The strain accumulated a significant level of RCII complexes on the one side and unassembled CP47 and CP43 on the other side indicating that the binding of both PSII antennae to RCII is impaired.

Re-sequencing of the GT-W and GT-P genome revealed one common mutation in a 50S ribosomal protein L3 in comparison with the inferred chromosome sequence of the glucose-tolerant Williams strain. GT-W contained four specific mutations: one silent in the hypothetical protein Sll1367, the second in the PSII protein CP47, the third in the 500 kDa protein Slr0364, and the fourth in the long chain fatty acid coenzyme A-ligase (Slr1609; Table 1). The mutation in CP47 was a conservative amino acid change E283Q in the region of a large lumenal loop of the protein important for the assembly of PSII (Haag et al., 1993). A large deletion was found in the huge Slr0364 protein homologous to a protein involved in swimming motility (McCarren and Brahamsha, 2007).

Unexpectedly, no common mutations were found between the GT-W strain and the GT-O strains (Morris et al., 2014) also originating from the Vermaas lab. Although, there is a 5-year difference between the times the strains left the lab, this does not explain why there is no overlap in mutations and why GT-W and GT-P share the *rpl3* mutation while it is absent in GT-O.

The GT-P strain also contained a mutation in the *slr1609* gene. Interestingly, *slr1609* is the most frequently mutated gene—six independent (different) mutations can be found in *Synechocystis* strains sequenced by us and others (Tichý, unpublished data). The reason for this variability is unknown as is the effect of mutations on the activity of the enzyme, which is needed for reuse of fatty acids released from lipids after the action of lipases (Kaczmarzyk and Fulda, 2010). We checked the composition of pigment protein complexes in the *slr1609* deletion mutant but there was no apparent effect of the missing enzyme (Komenda and Tichý, unpublished data).

Apart from the point mutations, the specific feature of the GT-W chromosome was the presence of the large tandem duplication. Genome analysis of eukaryotes revealed that a tandemly arrayed duplicates account for significant proportion of genes and that duplication events are expected to play an important role in evolution of eukaryotes (Rizzon et al., 2006). Although, it is not clear whether gene duplication is a major evolutionary driving force also in prokaryotes compared to horizontal gene transfer, small tandem duplications on a single gene level have been shown previously to complement a non-photosynthetic phenotype of a PSII mutant (Tichy and Vermaas, 2000). In the current experiment we have sequenced 12 *Synechocystis* strains, among them several mutants and their revertants, and we have found one more example of another duplication between two transposases responsible for complementation of a *Synechocystis* mutant (Tichý, unpublished data). It suggests that such genome rearrangements are happening regularly and that under favorable conditions they may get easily selected for.

We have shown that loss of the duplication is accompanied by an increase in the level of PSI and by restoration of proper assembly of PSII (Figure 7). This indicates that the impaired autotrophic phenotype of the GT-W strain is related to the duplication and not to the observed specific mutations. We do not know whether the duplication in the GT-W strain is actually segregated or if there are still chromosomes present without the duplication as there is no simple check to confirm the non-duplicated variant. However, from the fact that the growth of GT-W in the absence of glucose easily led to the loss of the duplication it seems that the GT-W strain contains chromosomes both with and without the duplication. We think that the duplication got selected for during prolonged growth on glucose and that the lower level of PSI and impaired assembly of PSII are advantageous under glucose-induced stress. When the conditions change to photoautotrophy, the ratio of chromosomes with and without the duplication in cells can gradually change, finally leading to complete loss of the duplication.

We do not know how the duplication resulted in low levels of Chl and the inability of CP47 and CP43 to bind to RC in GT-W. Most of the genes present in the duplication code for proteins with unknown function, however, the presence of the gene coding for the PSII structural protein D1 is striking. We believe that the presence of an extra copy of this highly expressed gene could change the stoichiometry of subunits in this multi-subunit complex leading to the observed lower accumulation of PSII and higher accumulation of PSII intermediates. Such gene imbalances and their consequences caused by partial genome duplications have been extensively studied in eukaryotes (Birchler and Veitia, 2012). It has been shown that both under-expression and over-expression of protein complex subunits could lead to similar deleterious effects (Papp et al., 2003). Interestingly, in plants, genes coding for PSII subunits were mostly eliminated from chromosome regions produced by partial genome duplications as opposed to whole-genome duplications (Coate et al., 2011). This is explained by PSII being particularly gene-dosage sensitive (Coate et al., 2011), as incompletely assembled PSII not only impairs photosynthesis but also increase photo-oxidative damage in the cell (Komenda and Masojídek, 1995).

An extra gene copy of Psb28, which has been proposed to affect Chl biosynthesis (Dobáková et al., 2009), and Cycl (Slr1214), cyclase involved in the formation of the fifth ring of Chl, resulted in increased expression and accumulation of these proteins (Figure 7). However, this also could lead to an imbalance between individual enzymes of the Chl biosynthesis pathway envisioned to form a multi-enzymatic complex (Sobotka, 2014) Also there was more HemH, which might at least partly explain the low level of PP_*IX*_ detected in GT-W. Indeed, after the loss of duplication, the GT-Wrev showed a lower level of all of these proteins.

Specifically for prokaryotic cyanobacteria, where transcription is spatially connected to translation, it is also possible that the duplication disturbed the local arrangement in the PSII biogenesis centers further impairing correct and prompt PSII assembly and/or proper interaction with Chl biosynthetic machinery.

It is interesting that the GT-W background is appropriate for complete segregation of mutants in the Chl biosynthesis pathway which cannot segregate in the GT-P background. As an example we can mention the mutant lacking Ycf54, a protein factor needed for the efficient formation of the fifth ring during Chl biosynthesis (this volume of the journal). As mentioned above, the duplication limits the stress imposed on the mutant cells during segregation on glucose media and this helps them to survive mutations, which would be lethal in the other WT variants. On the other hand, rather small phenotypic changes in Chl biosynthesis caused by mutagenesis of GT-P can be enhanced by construction of the mutants in GT-W with already impaired Chl biosynthesis as demonstrated for the Psb28-less strain. As a consequence, the mutants show better detectable phenotypic differences which helps identify the function of proteins encoded by the mutated genes. In conclusion, this study shows that the choice of the proper background strain is not only essential for correct evaluation of mutant phenotypic characteristics but also for successful construction of severely affected mutants that might not survive when made in the inappropriate WT variant.

## Author contributions

MT performed experiments, evaluated and interpreted data, wrote the manuscript, MB performed experiments and evaluated data, JKop performed experiments, JN performed experiments, evaluated and interpreted data, wrote the manuscript, RS performed experiments, evaluated, and interpreted data, wrote the manuscript, JK performed experiments, evaluated, and interpreted data, wrote the manuscript.

## Funding

The work was supported by National Programme of Sustainability I of The Ministry of Education, Youth and Sports, ID: LO1416.

### Conflict of interest statement

The authors declare that the research was conducted in the absence of any commercial or financial relationships that could be construed as a potential conflict of interest.

## References

[B1] Birchler J. A. Veitia R. A. (2012). Gene balance hypothesis: connecting issues of dosage sensitivity across biological disciplines. Proc. Natl. Acad. Sci. U.S.A. 109, 14746–14753. 10.1073/pnas.1207726109 22908297 PMC3443177

[B2] Boehm M. Romero E. Reisinger V. Yu J. Komenda J. Eichacker L. A.. (2011). Investigating the early stages of photosystem II assembly in *Synechocystis* sp. PCC 6803: isolation of CP47 and CP43 complexes. J. Biol. Chem. 286, 14812–14819. 10.1074/jbc.M110.207944 21339295 PMC3083219

[B3] Boehm M. Yu J. Reisinger V. Beckova M. Eichacker L. A. Schlodder E.. (2012). Subunit composition of CP43-less photosystem II complexes of *Synechocystis* sp PCC 6803: implications for the assembly and repair of photosystem II. Philos. Trans. R. Soc. Lond. B Biol. Sci. 367, 3444–3454. 10.1098/rstb.2012.0066 23148271 PMC3497071

[B4] Coate J. E. Schlueter J. A. Whaley A. M. Doyle J. J. (2011). Comparative evolution of photosynthetic genes in response to polyploid and nonpolyploid duplication. Plant Physiol. 155, 2081–2095. 10.1104/pp.110.169599 21289102 PMC3091097

[B5] Dobáková M. Sobotka R. Tichý M. Komenda J. (2009). Psb28 protein is involved in the biogenesis of the Photosystem II Inner Antenna CP47 (PsbB) in the Cyanobacterium *Synechocystis* sp PCC 6803. Plant Physiol. 149, 1076–1086. 10.1104/pp.108.130039 19036835 PMC2633855

[B6] Ferreira K. N. Iverson T. M. Maghlaoui K. Barber J. Iwata S. (2004). Architecture of the photosynthetic oxygen-evolving center. Science 303, 1831–1838. 10.1126/science.1093087 14764885

[B7] Guskov A. Kern J. Gabdulkhakov A. Broser M. Zouni A. Saenger W. (2009). Cyanobacterial photosystem II at 2.9-angstrom resolution and the role of quinones, lipids, channels and chloride. Nat. Struct. Mol. Biol. 16, 334–342. 10.1038/nsmb.1559 19219048

[B8] Haag E. Eaton-Rye J. J. Renger G. Vermaas W. F. J. (1993). Functionally important domains of the large hydrophilic loop of CP47 as probed by oligonucleotide-directed mutagenesis in Synechocystis sp. PCC 6803. Biochemistry 32, 4444–4454. 10.1021/bi00067a037 8476869

[B9] Jordan P. Fromme P. Witt H. T. Klukas O. Saenger W. Krauss N. (2001). Three-dimensional structure of cyanobacterial photosystem I at 2.5 A resolution. Nature 411, 909–917. 10.1038/35082000 11418848

[B10] Kaczmarzyk D. Fulda M. (2010). Fatty acid activation in cyanobacteria mediated by acyl-acyl carrier protein synthetase enables fatty acid recycling. Plant Physiol. 152, 1598–1610. 10.1104/pp.109.148007 20061450 PMC2832271

[B11] Kearse M. Moir R. Wilson A. Stones-Havas S. Cheung M. Sturrock S.. (2012). Geneious Basic: an integrated and extendable desktop software platform for the organization and analysis of sequence data. Bioinformatics 28, 1647–1649. 10.1093/bioinformatics/bts199 22543367 PMC3371832

[B12] Knoppová J. Sobotka R. Tichý M. Yu J. Konik P. Halada P.. (2014). Discovery of a chlorophyll binding protein complex involved in the early steps of photosystem II assembly in Synechocystis. Plant Cell 26, 1200–1212. 10.1105/tpc.114.123919 24681620 PMC4001378

[B13] Komenda J. Knoppová J. Kopečná J. Sobotka R. Halada P. Yu J. F.. (2012a). The Psb27 assembly factor binds to the CP43 complex of photosystem II in the Cyanobacterium *Synechocystis* sp PCC 6803. Plant Physiol. 158, 476–486. 10.1104/pp.111.184184 22086423 PMC3252115

[B14] Komenda J. Masojídek J. (1995). Structural changes of Photosystem II complex induced by high irradiance in cyanobacterial cells. Eur. J. Biochem. 233, 677–682. 10.1111/j.1432-1033.1995.677_2.x 7588816

[B15] Komenda J. Sobotka R. Nixon P. J. (2012b). Assembling and maintaining the Photosystem II complex in chloroplasts and cyanobacteria. Curr. Opin. Plant Biol. 15, 245–251. 10.1016/j.pbi.2012.01.017 22386092

[B16] Kopečná J. Komenda J. Bučínská L. Sobotka R. (2012). Long-Term acclimation of the cyanobacterium *Synechocystis* sp PCC 6803 to high light is accompanied by an enhanced production of chlorophyll that is preferentially channeled to trimeric Photosystem I. Plant Physiol. 160, 2239–2250. 10.1104/pp.112.207274 23037506 PMC3510144

[B17] Kopečná J. Pilný J. Krynická V. Tomčala A. Kis M. Gombos Z.. (2015). Lack of phosphatidylglycerol inhibits chlorophyll biosynthesis at multiple sites and limits chlorophyllide reutilization in *Synechocystis* sp. strain PCC 6803. Plant Physiol. 169, 1307–1317. 10.1104/pp.15.01150 26269547 PMC4587476

[B18] McCarren J. Brahamsha B. (2007). SwmB, a 1.12-megadalton protein that is required for nonflagellar swimming motility in Synechococcus. J. Bacteriol. 189, 1158–1162. 10.1128/JB.01500-06 17158680 PMC1797281

[B19] Morris J. Crawford T. Jeffs A. Stockwell P. Eaton-Rye J. Summerfield T. (2014). Whole genome re-sequencing of two ‘wild-type’strains of the model cyanobacterium *Synechocystis* sp. PCC 6803. New Zeal. J. Bot. 52, 36–47. 10.1080/0028825X.2013.846267

[B20] Nixon P. J. Michoux F. Yu J. F. Boehm M. Komenda J. (2010). Recent advances in understanding the assembly and repair of photosystem II. Ann. Bot. 106, 1–16. 10.1093/aob/mcq059 20338950 PMC2889791

[B21] Papp B. Pál C. Hurst L. D. (2003). Dosage sensitivity and the evolution of gene families in yeast. Nature 424, 194–197. 10.1038/nature01771 12853957

[B22] Porra R. J. Thompson W. A. Kriedemann P. E. (1989). Determination of accurate extinction coefficients and simultaneous equations for assaying chlorophylls *a* and *b* extracted with four different solvents: verification of the concentration of chlorophyll standards by atomic absorption spectroscopy. BBA-Bioenergetics 975, 384–394. 10.1016/S0005-2728(89)80347-0

[B23] Rippka R. Deruelles J. Waterbury J. B. Herdman M. Stanier R. Y. (1979). Generic assignments, strain histories and properties of pure cultures of cyanobacteria. J. Gen. Microbiol. 111, 1–61. 10.1099/00221287-111-1-1

[B24] Rizzon C. Ponger L. Gaut B. S. (2006). Striking similarities in the genomic distribution of tandemly arrayed genes in *Arabidopsis* and rice. PLoS Comp. Biol. 2:e115. 10.1371/journal.pcbi.0020115 16948529 PMC1557586

[B25] Roose J. L. Wegener K. M. Pakrasi H. B. (2007). The extrinsic proteins of photosystem II. Photosynth. Res. 92, 369–387. 10.1007/s11120-006-9117-1 17200881

[B26] Sakata S. Mizusawa N. Kubota-Kawai H. Sakurai I. Wada H. (2013). Psb28 is involved in recovery of photosystem II at high temperature in *Synechocystis* sp. PCC 6803. BBA-Bioenergetics 1827, 50–59. 10.1016/j.bbabio.2012.10.004 23084968

[B27] Sobotka R. (2014). Making proteins green; biosynthesis of chlorophyll-binding proteins in cyanobacteria. Photosynth. Res. 119, 223–232. 10.1007/s11120-013-9797-2 23377990

[B28] Tichy M. Vermaas W. (2000). Combinatorial mutagenesis and pseudorevertant analysis to characterize regions in loop E of the CP47 protein in *Synechocystis* sp. PCC 6803. Eur. J. Biochem. 267, 6296–6301. 10.1046/j.1432-1327.2000.01718.x 11012684

[B29] Umena Y. Kawakami K. Shen J. R. Kamiya N. (2011). Crystal structure of oxygen-evolving photosystem II at a resolution of 1.9 A. Nature 473, 55–60. 10.1038/nature09913 21499260

[B30] Williams J. G. K. (1988). Construction of specific mutations in Photosystem-II photosynthetic reaction center by genetic-engineering methods in *Synechocystis*-6803. Method. Enzymol. 167, 766–778. 10.1016/0076-6879(88)67088-1

