# Supplementary material for: Strain of Synechocystis PCC 6803 with Aberrant Assembly of Photosystem II Contains Tandem Duplication of a Large Chromosomal Region
Source: Front Plant Sci. 2016 May 12;7:648. doi: 10.3389/fpls.2016.00648 (PMC4867675; doi:10.3389/fpls.2016.00648)
Supplement: Supplementary file 1 [file Table1.doc]

**Supplemental Table S1**. List of all genes located in the duplicated chromosomal segment in the WT-W strain. The order of genes reflects that in the chromosome.

| **Gene** | **Product** |
| --- | --- |
| ***sll0431*** | transposase |
| ***ssr0817*** | transposase |
| ***slr0462*** | transposase |
| ***sll0430*** | heatshock protein |
| ***slr1176*** | glucose-1-phosphateadenylyl transferase |
| ***slr1177*** | hypothetical protein |
| ***slr1178*** | hypothetical protein |
| ***slr1179*** | anhydro-N-acetylmuramic acid kinase |
| ***sll1110*** | peptide chain release factor |
| ***sll1109*** | hypothetical protein |
| ***sll1108*** | stationary-phase survival protein SurE homolog |
| ***slr1181*** | photosystem II D1 protein |
| ***slr1182*** | hypothetical protein |
| ***slr1183*** | hypothetical protein |
| ***slr1184*** | hypothetical protein |
| ***slr1185*** | cytochrome b6/f-complex iron-sulfur protein |
| ***slr1186*** | hypothetical protein |
| ***slr1187*** | hypothetical protein |
| ***slr1188*** | chloroplast membrane-associated 30 kD protein |
| ***slr1189*** | hypothetical protein |
| ***sll1107*** | hypothetical protein |
| ***sll1106*** | hypothetical protein |
| ***slr1192*** | zinc-containing alkohol dehydrogenase family |
| ***slr0585*** | argininosuccinate synthetase |
| ***slr0586*** | hypothetical protein |
| ***slr0587*** | hypothetical protein |
| ***sll0578*** | Phosphoribosylaminoimidazole carboxylase |
| ***sll0577*** | hypothetical protein |
| ***slr0588*** | hypothetical protein |
| ***slr0589*** | hypothetical protein |
| ***slr0590*** | hypothetical protein |
| ***sll0576*** | putative sugar-nucleotide epimerase/dehydratase |
| ***sll0575*** | ATP-binding protein |
| ***sll0574*** | probable lipopolysaccharide ABC transporter permease protein |
| ***slr0591*** | hypothetical protein |
| ***sll0573*** | carbamate kinase |
| ***sll0572*** | hypothetical protein |
| ***slr0592*** | hypothetical protein |
| ***slr0593*** | cAMP protein kinase regulatory chain |
| ***sll0569*** | recombinase A |
| ***slr0594*** | hypothetical protein |
| ***sll0567*** | Ferric uptake regulation protein |
| ***slr0597*** | Inosinemonophosphate cyclohydrolase |
| ***sll0565*** | hypothetical protein |
| ***sll0564*** | hypothetical protein |
| ***slr0598*** | hypothetical protein |
| ***slr0599*** | serine/threonine kinase |
| ***slr0600*** | hypothetical protein |
| ***slr0601*** | hypothetical protein |
| ***slr0602*** | hypothetical protein |
| ***sll0563*** | hypothetical protein |
| ***slr0603*** | DNA polymerase III alpha subunit |
| ***slr0604*** | LepA gene product |
| ***slr0605*** | hypothetical protein |
| ***slr0606*** | hypothetical protein |
| ***slr0607*** | hypothetical protein |
| ***slr0608*** | phosphoribosyl-ATP pyrophosphohydrolase |
| ***slr0609*** | cobalamin synthesis protein P47K/CobW |
| ***slr0610*** | hypothetical protein |
| ***slr0611*** | solanesyldiphosphate synthase |
| ***slr0612*** | hypothetical protein |
| ***slr0613*** | hypothetical protein |
| ***sll0558*** | hypothetical protein |
| ***sll1214*** | magnesium-protoporphyrin IX monomethyl ester cyclase |
| ***sll1213*** | hypothetical protein |
| ***sll1212*** | GDP-D-mannose dehydratase |
| ***slr1311*** | photosystem II D1 protein |
| ***slr1312*** | arginine decarboxylase |
| ***sll1209*** | NAD-dependent DNA ligaseLigA |
| ***slr1315*** | hypothetical protein |
| ***slr1316*** | iron(III) dicitrate transport system permease protein |
| ***slr1317*** | iron(III) dicitrate transport system permease protein |
| ***slr1318*** | iron(III) dicitrate transport system permease protein |
| ***slr1319*** | iron(III) dicitrate transport system permease protein |
| ***sll1206*** | ferric aerobactin receptor |
| ***sll1205*** | regulatory protein PchR |
| ***sll1204*** | hypothetical protein |
| ***sll1203*** | hypothetical protein |
| ***sll1202*** | hypothetical protein |
| ***sll1409*** | ferrichrome-iron receptor |
| ***sll1408*** | regulatory protein PcrR |
| ***sll1407*** | hypothetical protein |
| ***sll1406*** | ferrichrome-iron receptor |
| ***sll1405*** | biopolymer transport ExbD protein homologue |
| ***sll1404*** | biopolymer transport ExbB protein homologue |
| ***slr1484*** | hypothetical protein |
| ***slr1488*** | ABC transporter |
| ***slr1489*** | regulatory protein PchR |
| ***slr1490*** | ferrichrome-iron receptor |
| ***slr1491*** | iron(III) dicitrate-binding periplasmic protein |
| ***slr1492*** | iron(III) dicitrate-binding periplasmic protein |
| ***slr1493*** | hypothetical protein |
| ***slr1494*** | ABC transporter |
| ***sll1401*** | hypothetical protein |
| ***sll1400*** | hypothetical protein |
| ***ssl2733*** | hypothetical protein |
| ***sll1399*** | hypothetical protein |
| ***sll1398*** | photosystem II 13 kD protein Psb28 |
| ***slr1495*** | hypothetical protein |
| ***sll1397*** | transposase |
