# Supplementary material for: Strain of Synechocystis PCC 6803 with Aberrant Assembly of Photosystem II Contains Tandem Duplication of a Large Chromosomal Region
Source: Front Plant Sci. 2016 May 12;7:648. doi: 10.3389/fpls.2016.00648 (PMC4867675; doi:10.3389/fpls.2016.00648)
Supplement: Supplementary file 2 [file Image1.PDF]

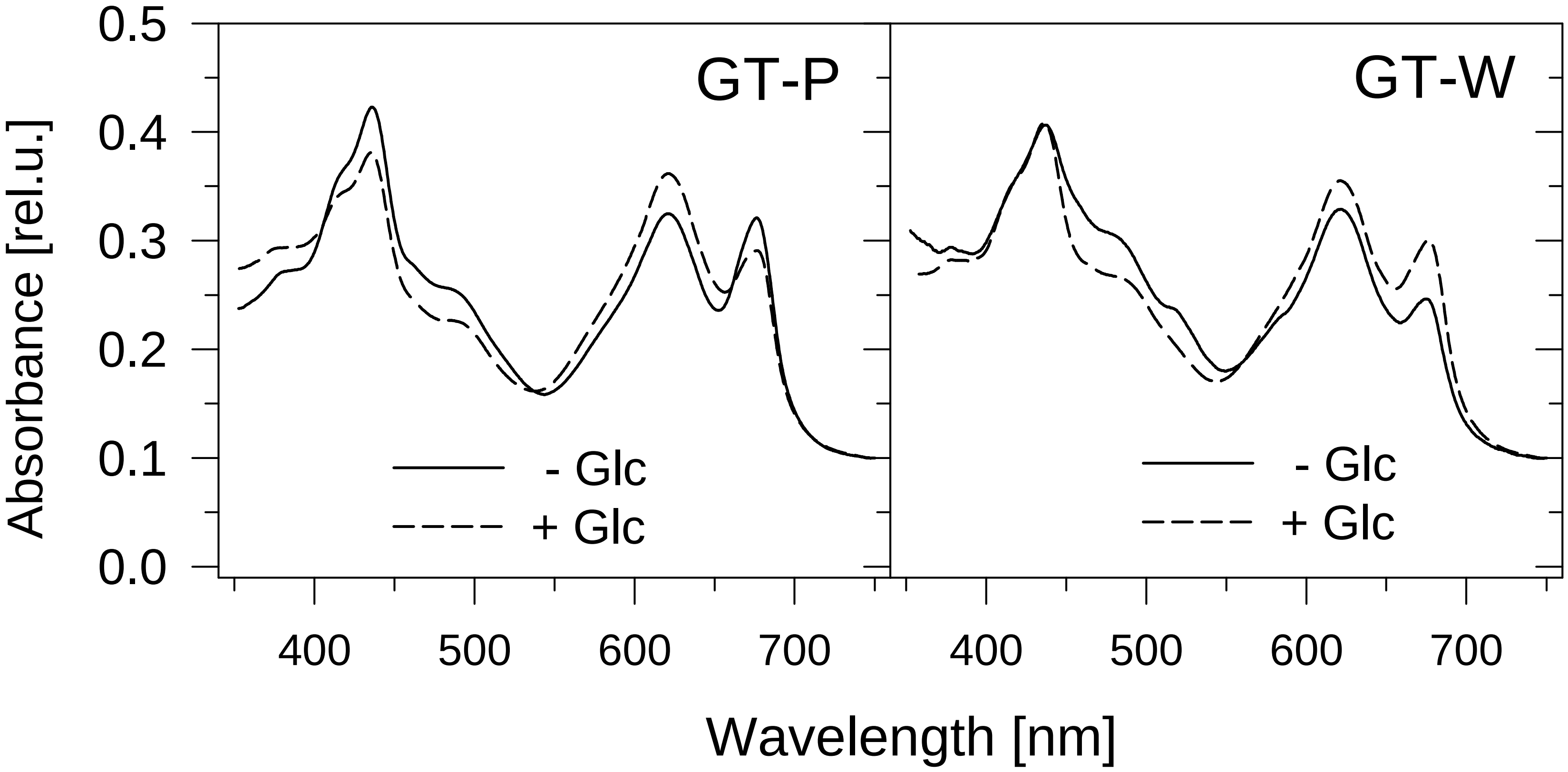

Fig. S1. Whole cell absorption spectra of the GT-P and GT-W strains grown in the absence and presence of 5 mM glucose. The spectra were measured by Shimadzu UV3000 spectrophotometer and were normalized for absorbance of 0.1 at 750 nm.
