# Supplementary material for: Strain of Synechocystis PCC 6803 with Aberrant Assembly of Photosystem II Contains Tandem Duplication of a Large Chromosomal Region
Source: Front Plant Sci. 2016 May 12;7:648. doi: 10.3389/fpls.2016.00648 (PMC4867675; doi:10.3389/fpls.2016.00648)
Supplement: Supplementary file 3 [file Image2.pdf]

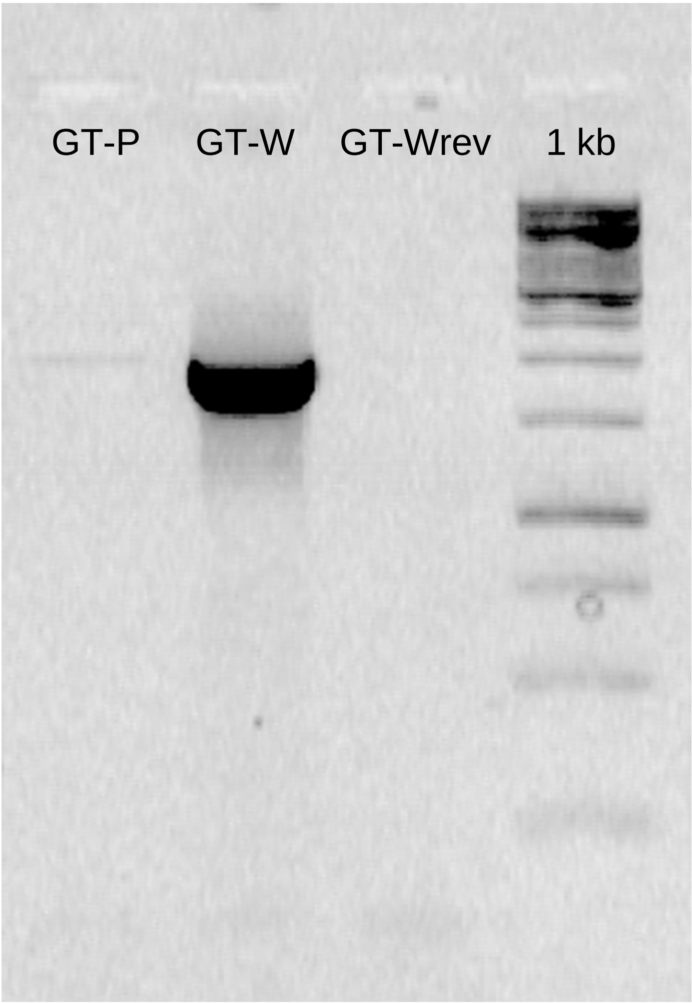

Fig. S2. PCR detection of the 110 kbp duplication using outbound primers from the beginning and from the end of duplication. The expected size of the PCR product is 1690 bp. The size of the closest bands on the 1 kb DNA ladder is 1.5 and 2 kbp.
